# Evaluation of Psychometric Properties and Factorial Structure of ADHD Module of K-SADS-PL in Children From Rural Kenya

**DOI:** 10.1177/1087054717753064

**Published:** 2018-02-02

**Authors:** Symon M. Kariuki, Charles R. J.C. Newton, Amina Abubakar, Mary A. Bitta, Rachael Odhiambo, Jacqueline Phillips Owen

**Affiliations:** 1 KEMRI-Wellcome Trust Research Programme, Kilifi, Kenya; 2 Department of Psychiatry, University of Oxford, UK; 3 Pwani University, Kilifi, Kenya; 4 Aga Khan University, Nairobi, Kenya; 5 King’s College London, UK; 6 South London and The Maudsley NHS Foundation Trust, UK

**Keywords:** K-SADS-PL, attention deficit hyperactivity disorders, psychometric properties, children, Kenya

## Abstract

**Objective:** We determined the reliability of The Kiddie-Schedule for Affective Disorders and Schizophrenia for School-Age Children–Present and Lifetime (K-SADS-PL) for screening and diagnosing ADHD in children. **Method:** K-SADS-PL was administered to 2,074 children in the community. Psychometric properties, factorial structure, and clinical validity of K-SADS-PL in screening or diagnosis of ADHD were examined. **Results:** Internal consistency was excellent for items in the screening interview (Macdonald’s Omega [ω] = 0.89; 95% confidence interval [CI] [0.87, 0.94]) and diagnostic supplement (ω = 0.95; 95% CI [0.92, 0.99]). The standardized coefficients for items in the screening interview were acceptable (0.59-0.85), while fit indices for single factorial structure reached acceptable levels. Screening items were associated with high sensitivity (97.8%; 95% CI [97.2, 98.5%]) and specificity (94.0%; 95% CI [93.0, 95.0%]) for diagnosis of ADHD in the supplement. The test-retest and interinformant reliability as measured by intraclass correlation coefficient was good for most of the items. **Conclusion:** This large study shows that K-SADS-PL can be reliably used to screen and diagnose ADHD in children in Kenya.

## Introduction

ADHD is one of the most common neuropsychiatric disorders in childhood that can persist into adulthood. According to the Global Burden of Disease, ADHD was responsible for 500,000 disability adjusted life years (DALYS), which is 0.2% of all mental health disorders (Erskine et al., 2014; Whiteford, Ferrari, Degenhardt, Feigin, & Vos, 2015). In spite of the high incidence of potential nongenetic risk factors for ADHD (particularly perinatal, infectious, and environmental factors) in many low- and middle-income countries (LMIC), there are no epidemiological studies on the burden of the condition in sub-Saharan Africa. This is ascribed to lack of child and adolescent psychiatrists and reliable assessment/diagnostic tools in many LMIC. Recent studies however show that researchers from LMIC countries, in collaboration with experts from high-income countries (HIC), can successfully adapt, validate, and apply available assessment/diagnostic tools (Abubakar et al., 2016; Kariuki, Abubakar, Murray, Stein, & Newton, 2016).

The Kiddie-Schedule for Affective Disorders and Schizophrenia for School-Age Children–Present and Lifetime (K-SADS-PL) version is popular for epidemiological and clinical studies of neuropsychiatric disorders in children, as it is regarded as a gold standard for *Diagnostic and Statistical Manual of Mental Disorders DSM-5* (5th ed.; *DSM-V*; American Psychiatric Association, 2013) diagnosis of ADHD (Kaufman et al., 1997). It is a semistructured questionnaire that assesses current and lifetime presence of affective and other psychiatric disorders, by interviewing both parents and children. It is validated across many settings particularly in HIC and found to possess good to excellent reliability and validity across a range of psychiatric disorders, including ADHD following validation in Israel (Shanee, Apter, & Weizman, 1997), Korea (Kim et al., 2004), Persia (Ghanizadeh, Mohammadi, & Yazdanshenas, 2006), and other HIC (Birmaher et al., 2009). However, most of the psychometric reports of K-SADS-PL have focused on all psychiatric conditions, with few reports of detailed evaluation of single syndromes such as ADHD, with some based only on clinical samples, which may be biased toward severe mental disorders (Kim et al., 2004). Additionally, none of these psychometric and clinical validity studies are from Africa, where the prevalence of ADHD is unknown, but may be common, according to recent studies of behavioral disorders in young children (Kariuki et al., 2016).

We evaluated the psychometric properties, reliability, and validity of ADHD module of K-SADS-PL in screening and diagnosing ADHD in epidemiological studies conducted in Africa. In particular, we aimed to examine the item reliability (including standardized coefficients/loadings); compute fit indices for the one-dimension K-SADS-PL; assess polychoric correlations, sensitivity/specificity/positive predictive values (PPV)/negative predictive values (NPV) of the screen interviews with diagnostic supplements; and determine test-retest and interrater reliability.

## Method

### Study Settings

This study was conducted on a rural area of Kenya in a demographic surveillance area (Scott et al., 2012). Fieldworkers on motorbikes visit every homestead every 4 months to update vital statistics on births, deaths, and migration patterns in this area. The main population within the study area are of the larger Mijikenda ethnic group, the majority of whom are subsistence farmers and a few fishermen. The literacy levels are low and this area is one of the poorest administrative regions in Kenya. The health care system for mental and neurodevelopmental disorders in this area is poorly developed (Bitta, Kariuki, Chengo, & Newton, 2017).

### Sampling

All children aged 6 to 9 years living within demographic area, who form a total population of about 28,000 were eligible. This current study was nested in a large epidemiological study which aimed at screening about 15,000 children randomly selected from the 28,000 children aged 6 to 9 years within the demographic. The RAND() command of MySQL was used to randomly select the eligible children. The age group of 6 to 9 years was chosen because this is when most neurodevelopmental disorders such as ADHD become apparent. In this baseline epidemiological study, 11,223 children were successfully screened with neurodevelopmental disorders screening tool (NDST), and 2,162 invited for further clinical and neuropsychological assessments including administration of the K-SADS-PL. During piloting, NDST screened ADHD with a sensitivity of 89.2% (95% CI [88.7%, 89.8%]) and a specificity of 94.8% (95% CI [94.4, 99.3%]).

### Description of ADHD Module of K-SADS-PL

ADHD module of K-SADS-PL is a semistructured interview with two components: a screening interview and diagnostic supplement (Kaufman et al., 1997). The screening interview focuses on four items, namely (a) “Difficulty sustaining attention”; (b) “Task or play activities,” “Easily distracted”; (c) “Difficulty remaining seated”; and (d) “Impulsivity.” Children who reach a threshold score of 3 in the relevant subsection of the screening component have the K-SADS-PL supplement administered. The supplement has 4 categories of ADHD based on the *DSM-5* criteria: (a) predominantly inattentive subtype; (b) predominantly hyperactive-impulsive sub-type; (c) combined type; and (d) other specified ADHD; all of which manifests either in the past (>6 months ago) or the present (≤6 months to present).

### Procedures

The ADHD module of K-SADS-PL was translated into the local language, Kiswahili, through a standardized forward and back translation process according to international guidelines (http://www.who.int/substance_abuse/research_tools/translation/en/). A panel approach was used to harmonize the translation. A team made up of a developmental psychologist, epidemiologists, and professionals (clinicians, linguist, and research assistants) fluent in English, Swahili, and familiar with the local culture were members of this harmonization team. All the tools were piloted to test their appropriateness in assessing neurodevelopment and adapted accordingly before use in the epidemiological survey. Translation, adaptation, and administration of K-SADS-PL was supervised by a developmental psychologist (A.A.); child and adolescent psychiatrist (J.P.O.); linguists fluent in English, Kiswahili, and Mijikenda; and neuroepidemiologists (S.K. and C.N.).

K-SADS-PL was administered by assessors trained by a consultant child and adolescent psychiatrist (J.P.O.), who actively supervised the assessors on a weekly basis so that difficulties in rating items and making decisions on diagnosis could be resolved. The child and adolescent psychiatrist has reliability approval for administering and supervising administration of K-SADS-PL from an approved K-SADS-PL trainer (who is also a consultant child and adolescent psychiatrist based at Kings’ College London).

Trained administrators of the tool first introduced the purpose of the K-SADS-PL to the parents and the child, including explaining the scoring requirements to move from screening interview to diagnostic supplement. Questions were asked to both the parent and the child, with the assessor integrating their responses. Although ADHD module of K-SADS-PL requires parent–child participation, we relied mostly on the parent’s or caretaker’s accounts of the child’s behavior. In the screening interview, a response was rated as either absent (coded as 1), subthreshold levels (coded as 2), or threshold levels (coded as 3). While in the diagnostic supplement, ratings were done on a scale of 0, 1, or 2, with 2 representing presence of ADHD symptoms. Opposition defiant disorders, anxiety disorders, and other neuropsychiatric disorders (not reported in this study) were also considered for differential diagnosis when diagnosing ADHD.

Two weeks after initial screening with ADHD module of K-SADS-PL, 29 children were randomly selected for readministration of the screening interviews of the tool to examine the test-retest reliability. Another 20 children with and without ADHD in the supplement ADHD module of K-SADS-PL were examined through direct clinical interviews and/or recorded videos by a child and adolescent psychiatrist who was blinded to the initial diagnosis made by trained administrators of K-SADS-PL.

### Ethical Considerations

Written informed consent for the study was obtained from parents of children who participated in this work.

### Statistical Analysis

All analysis was performed with either STATA (version 13, Stata Corp, TX, USA) or R statistical software (version 3.4.0 (2017-04-21)). The internal consistency or interitem correlation was computed as MacDonald’s Omega (ω), using the *psych* package of R, with their CIs estimated by bootstrapping for 1,000 iterations. Reliability measured by ω presents better estimates than that of Cronbach’s alpha (α) when the tool or test has asymmetrical items, violation of normality introduced by small samples, or missing tau-equivalence (congeneric) (Trizano-Hermosilla & Alvarado, 2016), and so the former (ω) were presented as the primary measures of reliability.

**Figure 1.**
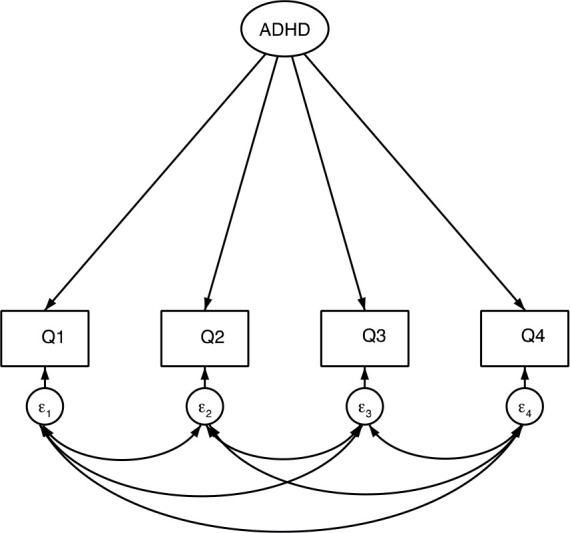
Outline of the one-dimensional factorial structure applied in computing fit indices for the screening and supplement ADHD modules of K-SADS-PL. *Note.* Q1-Q4 represents domains/items of ADHD, while ε_1_-ε_2_ represents the error terms that were subsequently allowed to interact. K-SADS-PL = Kiddie-Schedule for Affective Disorders and Schizophrenia for School-Age Children–Present and Lifetime.

Using structural equation models that assumed a linear trend for the three-ordinal responses of items of K-SADS-PL, we generated the standardized coefficients or loadings associated with responses from the screening interview and from the diagnostic supplement, while also investigating the fit indices (root mean squared error of approximation [RMSEA], Tucker–Lewis index [TLI], and comparative fit index [CFI]) associated with a one-dimensional factorial structure of the ADHD module of K-SADS-PL (Figure 1). We ensured models with modest fit by allowing the error terms with a modification index >10 from the structural model to correlate. The fit indices were considered acceptable if RMSEA was ≤0.05 and CFI and TLI was ≥0.90 (Browne & Cudeck, 1993; Kariuki et al., 2016). We examined screening and supplement responses using the polychoric correlations as the responses for either were ordinal. Using the diagnosis from the supplement interview as the reference standard, we estimated the sensitivities, specificities, PPV, and NPV associated with the screening interview ratings. Test-retest reliability (for 29 children rescreened 2 weeks later ensuing initial screening with ADHD module of K-SADS-PL) and interrater reliability (for 20 children reevaluated by a child and adolescent psychiatrist) was computed using the *irr* package in RR as intraclass correlation coefficients, specifying consistency types for test-retests, agreement types for interrater reliability, and one-way random effect to allow the estimates to vary across the participants. We also examined the overlap of ADHD with oppositional defiant disorders and anxiety disorders, using proportions and tetrachoric correlations and hypothesized that overlap will be more substantial in the former (which, like ADHD, is an externalizing disorder) than in the latter (which, unlike ADHD, is an internalizing disorder).

## Results

### General Description

Initial screen with ADHD K-SADS-PL module was done on 2,074 children, which is ~95% of those approached, following initial screening with NDST in Stage I. Of the 2,074 children aged 6 to 9 years, 1,084 (52.3%) were males. Those who reached the threshold in the initial screening were 385/2,074 (18.5%); response for individual questions is shown in Table 1. Of the 385 who screened positive, 298 (77.4%) further received supplement ADHD module of K-SADS-PL. ADHD was diagnosed in 285 who received the supplement, and as expected, there was low overlap with generalized anxiety disorder (3/285 [1.1%]; tetrachoric correlation ρ = .35, 95% CI [0.05, 0.64]), but substantial overlap with oppositional defiant disorders (72/285 [25.2%]; tetrachoric correlation ρ = .58, 95% CI [0.50, 0.65]).

**Table 1.** Proportion That Screened Positive for ADHD Symptoms in Each of the Four Domains of Screening and Supplement Module of K-SADS-PL.

Domains	ADHD K-SADS-PL module initial screen (*N* = 2,074)
K-SADS-PL screening	
Difficulty sustaining attention	252 (12.1%; 95% CI [10.8%, 13.6%])
Easily distracted/task or play activities	259 (12.4%; 95% CI [11.0%, 13.9%])
Difficulty remaining seated	328 (15.8%; 95% CI [14.2%, 17.4%])
Impulsivity	139 (6.7%; 95% CI [5.6%, 7.8%])
K-SADS-PL supplement	ADHD K-SADS-PL module supplement (*N* = 298)
Inattentive subtype	56 (18.7%; 95% CI [14.5%, 23.6%])
Hyperactive-impulsive subtype	76 (25.8%; 95% CI [20.9%, 31.2%])
Combined type	93 (31.2%; 95% CI [25.9%, 36.8%])
Other specified ADHD	43 (14.4%; 95% CI [10.6%, 18.4%])

*Note.* K-SADS-PL = Kiddie-Schedule for Affective Disorders and Schizophrenia for School-Age Children–Present and Lifetime; CI = confidence interval.

### Item Reliability Coefficients and Factorial Structure

The item reliability coefficients for the screening stage were excellent (McDonald’s Omega [ω] = 0.89; 95% CI [0.87, 0.94]). The items reliability coefficients for the supplement stage were excellent as well (ω = 0.95; 95% CI [0.92, 0.99]). The item reliability coefficients for K-SADS-PL screening items were comparable for males (ω = 0.89; 95% CI [0.72, 0.98]) and females (ω = 0.88; 95% CI [0.81, 0.92]).

The standardized coefficients associated with each item of the screening ADHD module of K-SADS-PL ranged from 0.59 to 0.85, with an overall mean of 0.75 (95% CI [0.56, 0.92]) (see Table 2). Most of the standardized coefficients for the supplement ADHD module of K-SADS-PL were statistically significant although they were lower than those for the screening stage (Table 2).

**Table 2.** Standardized Coefficients (Loadings) for the Domains of Screening and Supplement ADHD Module of K-SADS-PL in the Confirmatory Analysis (CFA).

Domains	Standardized coefficients (95% CI)
K-SADS-PL screening module	**Overall mean: 0.75 [0.56, 0.92]**
Difficulty sustaining attention	**0.77 [0.76, 0.78]**
Easily distracted/task or play activities	**0.77 [0.75, 0.78]**
Difficulty remaining seated/restlessness	**0.85 [0.84, 0.86]**
Impulsivity	**0.59 [0.56, 0.61]**
K-SADS-PL supplement module	**Overall mean: 0.15 [0.07, 0.23]**
Inattentive subtype	0.08 [–0.02, 0.19]
Hyperactive-impulsive subtype	**0.18 [0.07, 0.29]**
Combined type	**0.20 [0.09, 0.30]**
Other specified ADHD	**0.16 [0.05, 0.27]**

*Note.* Statistically significant standardized coefficients from the CFA are highlighted in bold. K-SADS-PL = Kiddie-Schedule for Affective Disorders and Schizophrenia for School-Age Children–Present and Lifetime; CFA = confirmatory factor analysis; CI = confidence interval.

Fit indices for the one-dimensional structure (outlined in Figure 1) were all acceptable for the screening ADHD module of K-SADS-PL (RMSEA = 0.00, TLI = 1.00, and CFI = 1.00). Similarly, the supplement ADHD module of K-SADS-PL produced excellent fit indices for the one-dimensional structure (RMSEA = 0.00, TLI = 1.04, and CFI = 1.00).

### Validity of the Screening Items

Rating for the screening item “difficulty sustaining attention” were correlated with ratings in all items in the ADHD supplement of K-SADS-PL, with two positive correlations (ρ = .37 for combined ADHD types and ρ = .17 for inattention ADHD type)) and two negative correlations (ρ = −0.17 for hyperactive/impulsive type and ρ = −0.17 for the unspecified ADHD type) (Table 3). The screening item “Easily distracted” was associated with all items in the ADHD supplement of K-SADS-PL; all were negative correlations (particularly with hyperactive/impulsive ADHD type (ρ = −.32), except one (ρ = .24 for combined type ADHD). Screening items difficulty remaining seated and impulsivity had the fewest significant polychoric correlations (Table 3).

**Table 3.** Polychoric Correlation of Ratings From Domains of Screening and Supplement ADHD Module of K-SADS-PL.

	Inattentive subtype	Hyperactive or impulsive subtype	Combined type	Other specified ADHD
Screening ADHD module of K-SADS-PL	ρ (95% CI)	ρ (95% CI)	ρ (95% CI)	ρ (95% CI)
Difficulty sustaining attention	**0.17 [0.01, 0.32]; *p* < .001**	**–0.28 [–0.41, –0.14]; *p* = .005**	**0.37 [0.23, –0.50]; *p* < .001**	**–0.17 [–0.32, –0.01]; *p* < .001**
Easily distracted/task or play activities	**–0.16 [–0.31, –0.01]; *p* < .001**	**–0.32 [–0.45, –0.18]; *p* < .001**	**0.24 [0.10, –0.37]; *p* <.001**	**–0.33 [–0.46, –0.49]; *p* = .006**
Difficulty remaining seated	**–0.05 [–0.24, –0.14]; *p* = .033**	0.14 [–0.03, 0.31]; *p* = .669	0.25 [0.07, 0.42]; *p* **=** .930	0.15 [–0.04, 0.34]; *p* = .398
Impulsivity	**–0.32 [–0.53, –0.10]; *p* < .001**	0.01 [–0.14, –0.16]; *p* < .001	0.12 [–0.03, –0.27]; *p* **<** .001	−0.50 [–0.61, 0.38]; *p* = 0.096

*Note.* The Polychoric correlations that are in bold are those whose lower confidence estimates did not cross below zero (for positive correlations) or above zero (for negative correlations). The likelihood ratio *p* values of goodness-of-fit test are also provided. K-SADS-PL = Kiddie-Schedule for Affective Disorders and Schizophrenia for School-Age Children–Present and Lifetime; CI = confidence interval.

Reaching a threshold score of 3 points in the relevant subsection of the screening component was associated with very high sensitivity (97.8%; 97.2%-98.5%) and specificity (94.0%; 93.0%-95.0%) for diagnosis of ADHD in the supplement stage, with NPV being higher than PPV, as expected of diagnostic tools (Table 4). As expected, individual screening items had the very high specificities (94.0%-98.2%), but as expected lower sensitivities (39.3%-82.2%) (Table 4).

**Table 4.** Sensitivity and Positive Predictive Values of Domains From Screening ADHD Module of K-SADS-PL Against a Diagnosis of Any ADHD From the Supplement ADHD Module of K-SADS-PL.

Screening ADHD module of K-SADS-PL	Sensitivity (95% CI)	Specificity (95% CI)	PPV	NPV
Positive for any question	97.8% [97.2%, 98.5%]	94.0% [93.0%, 95.0%]	72.4% [70.5%, 74.3%]	99.6% [99.3%, 99.9%]
Difficulty sustaining attention	63.1% [61.0%, 65.2%]	95.9% [95.1%, 96.8%]	71.7% [69.7%, 73.6%]	94.1% [93.1%, 95.2%)]
Easily distracted/task or play activities	64.2% [62.1%, 66.2%]	95.7% [94.9%, 96.6%]	70.9% [68.9%, 72.8%]	94.3% [93.3%, 95.3%]
Difficulty remaining seated	82.2% [81.1%, 84.4%]	94.8% [93.9%, 95.8%]	72.1% [70.2%, 74.1%]	97.1% [96.4%, 97.8%]
Impulsivity	39.3% [37.2%, 41.4%]	98.4% [97.8%, 98.9%]	79.8% [78.1%, 81.5%]	91.0% [89.7%, 92.2%]

*Note.* K-SADS-PL = Kiddie-Schedule for Affective Disorders and Schizophrenia for School-Age Children–Present and Lifetime; CI = confidence interval; PPV = positive predictive values; NPV = negative predictive values.

### Reliability of the ADHD Module of K-SADS-PL

Test-retest reliability was highest for the screening item “Easily distracted” both in one-way random effect model and in the two-way random effect model. The test-retest reliability was good for all other items (ICC ranged from 0.66-0.68; *p* < .001), except for impulsivity (Table 5). Similarly, the interrater reliability was highest for combined ADHD types both one-way random effect model (0.77; 95% CI [0.29, 0.95]) and two-way random effect model (0.77; 95% CI [0.29, 0.95]). The interrater reliability was good for all other items (ICC ranged from 0.64-0.76; *p* < .001), except for impulsivity (Table 6).

**Table 5.** Test-Retest for K-SADS-PL Screening Domains Administered in Stage 1 of the Study.

	Test-retest: One-way random effect model^a^	
K-SADS-PL screening subdomain	ICC consistency type (95% CI)	*p* value
Easily distracted/task or play activities	0.82 [0.66, 0.91]	8.2×10^−9^
Difficulty sustaining attention	0.68 [0.43, 0.83]	1.1×10^−5^
Impulsivity	0.20 [0, 0.52]	0.152
Difficulty remaining seated/restlessness	0.66 [0.40, 0.83]	2.4×10^−5^

*Note.* K-SADS-PL = Kiddie-Schedule for Affective Disorders and Schizophrenia for School-Age Children–Present and Lifetime; ICC = intraclass correlation; CI = confidence interval.

a All but one K-SADS-PL domains are very stable two weeks after the initial administration.

**Table 6.** Interrater Reliability for K-SADS-PL Supplement used in the Study.

	Interrater reliability: One way-random effect model^a^	
K-SADS-PL supplement subdomain	ICC agreement type (95% CI)	*p* value
Inattentive sub-type	0.76 [0.31, 0.93]	2.3×10^−3^
Hyperactivity-impulsivity sub-type	0.41 [0, 0.82]	0.110
Combined type	0.77 [0.29, 0.95]	4.6×10^−3^
Any ADHD type^b^	0.64 [0.13, 0.89]	4.5×10^−3^

*Note.* K-SADS-PL = Kiddie-Schedule for Affective Disorders and Schizophrenia for School-Age Children–Present and Lifetime; ICC = intraclass correlation; CI = confidence interval.

a All but one K-SADS-PL domains had high interrater reliability with the psychiatrist’s diagnosis.

b Any ADHD type is the overall category that covers even those other subcategories and that the interrater reliability was between neuropsychological assessors against a child and adolescent psychiatrist.

## Discussion

Findings from this large study show that K-SADS-PL can be reliably used to screen and diagnose ADHD in children from sub-Saharan Africa. Briefly, the item reliability coefficients were excellent (>0.80 for MacDonald’s Omega) and so were most standardized coefficients and fit indices for a single factorial structure. Screening interview ratings correlated well with those of the diagnostic supplement, with very high sensitivities (~97%) and specificities (~95%). Additionally, most of the items had an acceptable test-retest and interrater reliability (up to 0.77). As expected, the ADHD symptoms showed little overlap with anxiety disorders (1%), and high overlap with oppositional defiant disorders (25%).

The findings in this study are similar to studies in other cultures. For instance, similar interrater reliability (0.77 in our study vs. 0.75) and positive screen rate (15.8% in our study vs. 15.2%) in the Korean study (Kim et al., 2004). The proportion of overlap with oppositional defiant disorder were comparable to literature estimates of ~30%, as it is thought these two conditions are often comorbid and may share a common psychopathology (Biederman, Newcorn, & Sprich, 1991). Low overlap with anxiety disorders is reassuring in that anxiety symptoms manifesting as fidgeting, for example, will not be substantially misclassified as ADHD in this area. The findings, including the similarities across studies, highlight the invariance nature of the diagnostic value of K-SADS-PL across many societies, including in sub-Saharan Africa. However, there were some differences, for instance, the higher interrater reliabilities in HIC compared to this study is probably due to (a) methodological differences (e.g., interrater reliability in our study was computed with ICC, which are more robust compared to kappa agreements); (b) economic disparities (e.g., diagnostic abilities/facilities were likely more readily available for the American study than our study); and (c) literacy level and cultural differences (where symptoms for ADHD may be perceived or understood differently across societies). Importantly, our study provides additional information on item reliability coefficients, interitem correlation reliability, and fit indices for single factorial structure (estimates were acceptable) of ADHD module of K-SADS-PL; these were not examined by many previous studies from other settings.

The internal consistency is an important measure of the relatedness of the individual items used to assess or diagnose ADHD (Tang, Cui, & Babenko, 2014). In our study, MacDonald’s Omega estimates were excellent and comparable between males and females, suggesting that the items in the screening interviews and diagnostic supplements homogenously measure a common construct, that is, ADHD (Tang et al., 2014). This conclusion was further supported by acceptable fit indices, all of which assumed a one-dimensional factorial structure for the K-SADS-PL in screening and diagnosing ADHD. The acceptable fit indices (despite being based on a 3-point response scale often associated with lower factor loadings; Yuh & Muthen, 2002) suggest the configural invariance of ADHD module of K-SADS-PL even when used in Kenya. As few studies in the past have examined these fit indices for single factorial structure of ADHD module of K-SADS-PL; future studies should attempt to replicate our findings.

Item scale reliability (as measured by standardized coefficients) was within acceptable ranges for all items in the screening interview. This is important in test administration as it means the questions are capturing what they are intended to measure. However, standardized coefficients were much lower in the diagnostic supplement, which is less detrimental as its administration is dependent on reaching a threshold on the screening interview, whose standardized coefficients were all acceptable. In the screening interview, impulsivity had the lowest standardized coefficients, implying that this term is either (a) not fully understood in this region or (b) not a common manifestation of ADHD in this area. Our findings tended to support the former possibility as polychoric correlations with unspecified ADHD (symptoms not fully described/understood by parents/children) were largest with screening for impulsivity.

We performed polychoric correlations to examine how well ratings for the screening interview domains related with those of the diagnostic supplement, and two screening domains showed consistent significant correlations with all ratings of the diagnostic supplement. These were “difficulty sustaining attention” and “easily distracted” which may be the traits of ADHD that are best understood by parents in this community. The directionality (positive or negative) of these correlations depends on the broad categories of ADHD in the supplement (either hyperactivity or attention-deficit) the screening item of interest is designed to assess. For example, “difficulty sustaining attention” assesses the ADHD category “attention-deficit” and not “hyperactivity,” and so is positively correlated with the former category of ADHD and negatively with the latter. However, to improve sensitivity of obtaining reliable estimates of ADHD in epidemiological studies, other two screening items should also be included as they correlated with at least one ADHD type from supplement.

The sensitivities and specificities of positivity to any screening interview questions are very high, implying that these questions may be useful in identifying children requiring clinical assessment and care particularly in LMIC, where resources are so limited that full administration of the ADHD module of K-SADS-PL can be logistically intensive. High sensitivities are also important in detecting ADHD in epidemiological studies. Sensitivity may depend on the number of questions in a tool or their ability to accurately describe the symptoms of a condition of interest such as ADHD. Low sensitivity can result in false negatives and thereby missed opportunities for management and targeted care, which can be avoided by ensuring that a tool has adequate items that accurately describe the symptoms, and that the questions are properly adapted during translation for use in the local population. Nevertheless, all screening questions should be asked, as individual questions had higher specificities as expected. The sensitivities may depend on the correct diagnosis of ADHD at the supplement; fortunately, interrater reliability against a child psychiatrist was good for most items. Test-retest reliability screening interview questions, as measured by ICC, showed that most of the questions were stable over time and may persist. Although low test-retest ICC may suggest that ADHD symptoms were transient, those for impulsivity warrants further work, as its interrater reliability was also low.

This is the largest study in Africa to evaluate the utility of K-SADS-PL in screening and diagnosing ADHD, having involved over 2,000 children. The participants were randomly drawn from the general population as part of an epidemiological survey, and is thus far much more representative than hospital samples. In addition to psychometric properties and validity, the article additionally examines internal consistency and the factorial structure of ADHD module of K-SADS-PL, which were not reported in most previous studies. However, the study has a small overlap with anxiety disorders, may lead to slight misclassification limitations, which could be addressed by screening for both conditions. The Child and Adolescent Psychiatrist was not able to interact directly with many children receiving K-SADS-PL and had to review recorded videos of these children. This is understandable since the psychiatrist practices in the United Kingdom, and travelled severally to the study area to train the administrators of K-SADS-PL.

## Conclusions

We conclude that K-SADS-PL can be reliably used in rural settings in Africa to screen and diagnose ADHD in children. Neuropsychological assessors and primary health care workers can be trained and supervised by child and adolescent psychiatrists to administer K-SADS-PL in rural Kenya. Further work would still be needed to improve community understanding of ADHD symptoms such as impulsivity.
